# Applications of Behavioral Change Theories and Models in Health Promotion Interventions: A Rapid Review

**DOI:** 10.3390/bs15050580

**Published:** 2025-04-25

**Authors:** Areti-Dimitra Koulouvari, Artemis Margariti, Evanthia Sakellari, Anastasia Barbouni, Areti Lagiou

**Affiliations:** Laboratory of Hygiene and Epidemiology, Department of Public and Community Health, School of Public Health, University of West Attica, 115 21 Athens, Greece; epi2231@uniwa.gr (A.M.); sakellari@uniwa.gr (E.S.); abarbouni@uniwa.gr (A.B.); alagiou@uniwa.gr (A.L.)

**Keywords:** health promotion, behavioral epidemiology, behavioral change theories and models

## Abstract

Health behavior change is considered to be central in health promotion, as it can amplify disease prevention and reduce morbidity and mortality at the individual, community, or population level. Behavioral epidemiology, an emerging field of epidemiology, emphasizes the development of integrated, theory-grounded, and evidence-based health promotion interventions. In this context, the present rapid review aims to explore and identify the application of behavioral change theories and models in health promotion interventions, which may address a wide range of topics and may target diverse population groups. The search was conducted in the PubMed and Scopus databases, following the PRISMA 2020 guidelines for scoping reviews. The selected studies were published between 2014 and 2024. A total of forty-five studies met the inclusion criteria. Most of the selected studies employed a combination of behavioral theories and/or models. Some studies were grounded in specific behavioral theories or models, while others developed emerging models. The results of this rapid review suggest that health promotion interventions grounded in behavioral theories/models indicate significant promise. However, further research is needed to pave the way for more effective and efficient health promotion interventions targeting in behavior change.

## 1. Introduction

Health behavior change is considered to be central in health promotion, as it can amplify disease prevention, reduce morbidity and mortality at individual, community or population level (R. Glasgow et al., 2004; Glanz et al., 2008; Davis et al., 2015; Davidson & Scholz, 2020; O’Connor, 2020; Willmott & Rundle-Thiele, 2021; Michaelsen & Esch, 2022; J. Wang et al., 2025). Specifically, the emerging field of behavioral epidemiology focuses on integrated health promotion interventions that are evidence-based and grounded in specific theories and models of behavior change (Sallis et al., 2000; Rubinelli & Diviani, 2020; Bauch et al., 2012; Raymond, 1989).

The significant relationship between health and behavior was first recognized in ancient Greece. Specifically, Hippocrates, in his work “Airs, Waters, and Places”, stated that “health is defined on the basis of a balance between environmental forces and individual habits” (Tountas, 2009). Nowadays, the connection between health and behavior remains central to the field of health promotion (Ochiai et al., 2021; Rubinelli & Diviani, 2020; Sallis et al., 2000). For example, in 1979, the Healthy People Initiative highlighted the extent to which behaviors and lifestyle patterns influence chronic disease outcomes in the United States (Ochiai et al., 2021). Since then, specialized fields within health sciences have emerged, focusing on the impact of behavior on prevention and health promotion (Helfer et al., 2020; Jenkins, 2003; Ochiai et al., 2021; Ory, 2002; Prochaska et al., 2008; Rubinelli & Diviani, 2020; Sallis et al., 2000).

Health promotion interventions and programs specifically target a wide range of behavioral outcomes, such as preventing and reducing health-risk or unhealthy behaviors, encouraging health-promoting behaviors, improving lifestyles, promoting the effective use of healthcare, and supporting the self-management of diseases (Lippke et al., 2012; Michie et al., 2018b; Noar et al., 2008; Prochaska et al., 2008). To achieve these aims, health promotion interventions utilize behavioral theories and models that offer a theoretical framework for explaining, assessing, predicting, and modifying behaviors (Davis et al., 2015; Kok et al., 2016; McCain, 2015; J. Wang et al., 2025). As Kurt Lewin mentioned, “There is nothing so practical as a good theory” (McCain, 2015), and so it is crucial to understand that the choice of a particular behavioral theory or model, along with the context in which it is applied, can vary and influence the effectiveness, reliability, and validity of the outcomes (Glanz et al., 2008; Glanz & Bishop, 2010; R. Glasgow et al., 2004; Lippke & Ziegelmann, 2008; Noar et al., 2008; Silva et al., 2024).

The evidence base for health promotion targeting in behavior change lacks sufficient information on the feasibility, cost-effectiveness, and evaluation of interventions, making it difficult to replicate these programs across diverse populations, conditions, and settings (Anderson et al., 2004; Becker et al., 2020; Cash et al., 2023; Collins et al., 2013; R. Glasgow et al., 2004; O’Connor, 2020; Prestwich et al., 2015; Rimer et al., 2001; J. Wang et al., 2025). Future efforts should focus on understanding the various behavioral health factors that influence healthy lifestyle patterns, identifying effective ways to implement strategies that promote and motivate health-enhancing behaviors, and reduce health-risk behaviors (Conner & Norman, 2017; Gardner et al., 2023; R. E. Glasgow & Emmons, 2007; Heino et al., 2021; Keller et al., 2021; Lippke et al., 2012; Morabia & Costanza, 2010). In this context, the present rapid review aims to explore and identify the application of behavioral change theories and models in health promotion interventions, which may address a wide range of topics and may target diverse population groups.

## 2. Methods

### 2.1. Study Design

A rapid review method was employed to explore and identify the application of behavioral change theories and models in health promotion interventions, which may address a wide range of topics and may target diverse population groups. This rapid review adhered to the necessary methodological strategy to yield sufficient results (Haby et al., 2024). The review process was carried out over eight months. The study was conducted following the Preferred Reporting Items for Systematic Reviews and Meta-Analyses (PRISMA) guidelines for scoping reviews, as outlined for the purposes of this study (Haby et al., 2024; Tricco et al., 2018).

### 2.2. Inclusion/Exclusion Criteria

The study selection criteria were based on the PICOS framework (Population, Interventions, Comparators, Outcomes, Study Design).

The inclusion criteria were the following:

(a) Studies published in English in peer-reviewed journals between January 2014 and April 2024;
(b) Studies with open access availability;
(c) Studies targeting various populations;
(d) Studies targeting a wide range of health promotion topics;
(e) Studies with sufficient and efficient reporting on methodological issues related to the choice and application of behavioral theories and models.

The exclusion criteria included the following:

(a) Studies not published in peer-reviewed journals;
(b) Studies not published in English;
(c) Studies with no open access availability.

In Table 1 the inclusion and exclusion criteria of the study characteristics based on the PICOS framework are summarized (da Costa Santos et al., 2007; Garritty et al., 2021).

### 2.3. Information Sources

To identify the publications for this rapid review, the authors searched the recent literature in the PubMed and Scopus databases between 2014 and 2024, οn 25 April 2024.

### 2.4. Search Strategy and Selection Process

The PRISMA guidelines for scoping reviews were applied in the search methodology (Haby et al., 2024; Tricco et al., 2018). The search terms used were as follows: “behavioral theor*” OR “behavior theor*” OR “behaviour theor*” OR “behavior change theor*” OR “behaviour change theor*” OR “behavioral model*” OR “behavior model*” OR “behaviour model*” OR “behavior change model*” OR “behaviour change model*” AND “behavior change” OR “behaviour change” AND “health promotion” AND “study protocol”. Regarding the review strategy, the first and second authors independently screened all titles and abstracts. Subsequently, full texts were reviewed to assess whether they met the inclusion criteria outlined above. Any conflicts that arose during the screening process were discussed between the two authors until a consensus was reached. Moreover, the tables and figures of the present rapid review were uploaded to OSF at the following link: https://osf.io/thwje/ (accessed on 16 April 2025).

### 2.5. Data Collection Process

The first and the second author collaborated to ensure data completeness and accuracy. Basic socio-demographic data as well as methodological data, related to exposures and outcomes, were extracted from each study. Additionally, the theoretical foundation of each study (e.g., application of a specific behavioral change theory or model) was intensely and thoroughly examined. Key information was extracted from the studies, including the health promotion topic, the study aim and focus, the level of influence according to McLeroy’s Ecological Model (intrapersonal/interpersonal/community level), the level of prevention (primary/secondary/tertiary level), the study design, the target group, the number of participants, and the intervention description (Table 2).

## 3. Results

### 3.1. Study Selection

**Figure 1:**
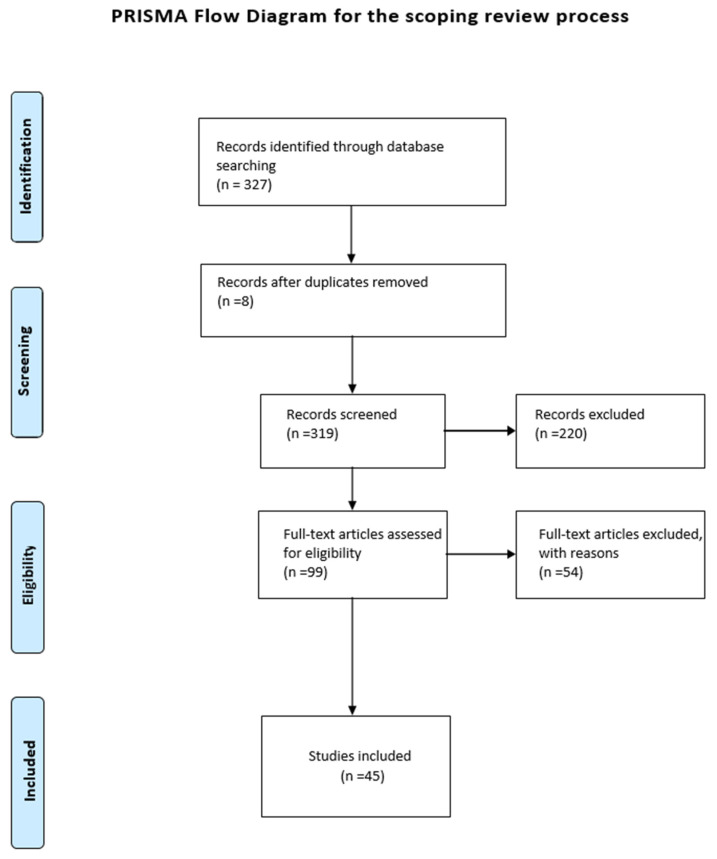
PRISMA flowchart search strategy.

A total amount of 327 abstracts were retrieved from both databases, specifically 46 records from PubMed and 281 from Scopus. Eight of these duplicates (n = 8) were excluded. The remaining 319 records were screened for eligibility based on the abstract, and 220 records were excluded since they did not fulfill the inclusion criteria: specifically, they did not provide sufficient and efficient reporting on methodological issues related to the choice and application of behavioral theories/models. Therefore, the 99 remaining records were screened for the full text, and 54 records were excluded due to the fact they did not meet the inclusion criteria: specifically, 44 records were not based on a specific behavioral change theory or model, three records concerned therapeutic methods, three records were at preliminary stage, two records had complex methodological approach which was considered to be beyond the scope of the present study, one record concerned only process evaluation, and one record concerned the development of a manual. Finally, 45 records were included in the present rapid review (Figure 1).

### 3.2. Study Design

Forty (88.88%) of the selected studies were study protocols for randomized controlled trials (RCTs) (Abidi et al., 2016; Abildsnes et al., 2017; Ara et al., 2019; Barrett et al., 2018; Bestle et al., 2020; Caudwell et al., 2016; C. Chen et al., 2020; W. Chen et al., 2016; Cleary et al., 2014; Conroy, 2022; Delea et al., 2019; Demetriou & Bachner, 2019; Dobson et al., 2016; Ek et al., 2018; Frost et al., 2022; Gardner et al., 2014; Godoy-Izquierdo et al., 2023; Gul et al., 2019; Haug et al., 2014; Jolly et al., 2018; Kamal et al., 2016; Krämer et al., 2022; Larkin et al., 2017; Larsen et al., 2020; Lubans et al., 2016; Lyon et al., 2024; Mâsse et al., 2020; Mat Said et al., 2021; Murawski et al., 2018; Nakamura et al., 2017; Pakpour et al., 2022; Patel et al., 2019; Perry et al., 2023; Pond et al., 2019; Rashid et al., 2022; Sanchez et al., 2024; Ting et al., 2018; Wallbank et al., 2019; K. Wang et al., 2022; Yoong et al., 2022). The remaining studies consisted of study protocols for: four controlled trials (8.88%), (Howlett et al., 2017; Lackinger et al., 2015; Latomme et al., 2021; Stea et al., 2016), and one single group intervention study (2.22%), (Freyer-Adam et al., 2022).

### 3.3. Behavioral Change Theory or Model

The use of a combination of behavioral change theories or models was more frequent (n = 28, 62.22%), (Abidi et al., 2016; Abildsnes et al., 2017; Caudwell et al., 2016; C. Chen et al., 2020; W. Chen et al., 2016; Conroy, 2022; Delea et al., 2019; Demetriou & Bachner, 2019; Ek et al., 2018; Gardner et al., 2014; Howlett et al., 2017; Jolly et al., 2018; Kamal et al., 2016; Larsen et al., 2020; Latomme et al., 2021; Lubans et al., 2016; Lyon et al., 2024; Mâsse et al., 2020; Mat Said et al., 2021; Nakamura et al., 2017; Patel et al., 2019; Perry et al., 2023; Pond et al., 2019; Rashid et al., 2022; Sanchez et al., 2024; Stea et al., 2016; Wallbank et al., 2019; Yoong et al., 2022).

Additionally, a specific behavioral change theory or model was applied as follows:

(a) Theory of Planned Behavior (n = 3, 6.66%), (Cleary et al., 2014; Larkin et al., 2017; Ting et al., 2018);
(b) Social Cognitive Theory (n = 3, 6.66%), (Barrett et al., 2018; Bestle et al., 2020; Murawski et al., 2018);
(c) Health Action Process Model (n = 3, 6.66%), (Godoy-Izquierdo et al., 2023; Haug et al., 2014; Krämer et al., 2022);
(d) Transtheoretical Model (n = 2, 4.44%), (Freyer-Adam et al., 2022; Pakpour et al., 2022);
(e) Emerging Models (n = 2, 4.44%), (Gul et al., 2019; Lackinger et al., 2015);
(f) Health Belief Model (n = 1, 2.22%), (K. Wang et al., 2022);
(g) Capability, Opportunity, Motivation Model (COM-B Model) (n = 1, 2.22%), (Frost et al., 2022);
(h) Behavior Change Communication (n = 1, 2.22%), (Ara et al., 2019);
(i) Behavior Change Techniques (n = 1, 2.22%), (Dobson et al., 2016).

### 3.4. Health Promotion Topics

The behavioral change theories or models were applied within health promotion interventions addressing a wide range of health promotion topics, as follows:

(a) Main Risk Factors (n = 27, 60%):
  - Physical activity (Barrett et al., 2018; C. Chen et al., 2020; Demetriou & Bachner, 2019; Ek et al., 2018; Gardner et al., 2014; Godoy-Izquierdo et al., 2023; Howlett et al., 2017; Lackinger et al., 2015; Larkin et al., 2017; Larsen et al., 2020; Latomme et al., 2021; Lubans et al., 2016; Murawski et al., 2018; Perry et al., 2023; Wallbank et al., 2019; Yoong et al., 2022);
  - Nutrition (Ara et al., 2019; Bestle et al., 2020; Jolly et al., 2018; Nakamura et al., 2017; Patel et al., 2019; Pond et al., 2019);
  - Physical activity and nutrition (Abildsnes et al., 2017);
  - Alcohol use (Abidi et al., 2016; Caudwell et al., 2016);
  - Alcohol and tobacco use (Haug et al., 2014);
  - Health behaviors (tobacco use, alcohol use, physical activity, nutrition) (Freyer-Adam et al., 2022).

(b) Prevention of Noncommunicable Diseases (n = 13, 28.88%):
  - Diabetes (Dobson et al., 2016; Ting et al., 2018);
  - Cardiovascular disease (Kamal et al., 2016; Mat Said et al., 2021; Sanchez et al., 2024);
  - Weight management/obesity (Mâsse et al., 2020; Rashid et al., 2022; Stea et al., 2016);
  - Frailty (Frost et al., 2022);
  - Sun protection (Cleary et al., 2014);
  - Postpartum contraception (Gul et al., 2019);
  - Oral health (K. Wang et al., 2022);
  - Protective behavior in the workplace (W. Chen et al., 2016).

(c) Mental Health Prevention and Promotion (n = 3, 6.66%):
  - Social, emotional, and behavioral development of school students (Lyon et al., 2024);
  - Depression (Krämer et al., 2022);
  - Internet gaming disorder (Pakpour et al., 2022).

(d) Prevention of Communicable Diseases (n = 2, 4.44%):
  - Sanitation (Delea et al., 2019);
  - COVID-19 (Conroy, 2022).

### 3.5. The Levels of Influence

The application of behavioral change theories or models in relation to levels of influence, based on McLeroy’s Socio-Ecological model, is presented as follows:

(a) Intrapersonal level (n = 27, 60%) (Abidi et al., 2016; Abildsnes et al., 2017; Ara et al., 2019; Caudwell et al., 2016; Cleary et al., 2014; Dobson et al., 2016; Ek et al., 2018; Freyer-Adam et al., 2022; Frost et al., 2022; Gardner et al., 2014; Godoy-Izquierdo et al., 2023; Gul et al., 2019; Haug et al., 2014; Howlett et al., 2017; Jolly et al., 2018; Lackinger et al., 2015; Larkin et al., 2017; Latomme et al., 2021; Lyon et al., 2024; Pakpour et al., 2022; Patel et al., 2019; Pond et al., 2019; Ting et al., 2018; Wallbank et al., 2019; K. Wang et al., 2022; Yoong et al., 2022);
(b) Intrapersonal and interpersonal levels (n = 9, 20%) (W. Chen et al., 2016; Conroy, 2022; Demetriou & Bachner, 2019; Kamal et al., 2016; Larsen et al., 2020; Mâsse et al., 2020; Mat Said et al., 2021; Nakamura et al., 2017; Rashid et al., 2022);
(c) Intrapersonal, interpersonal, and community levels (n = 5, 11.11%) (C. Chen et al., 2020; Delea et al., 2019; Lubans et al., 2016; Perry et al., 2023; Stea et al., 2016);
(d) Interpersonal level (n = 3, 6.66%) (Barrett et al., 2018; Bestle et al., 2020; Murawski et al., 2018);
(e) Intrapersonal and community levels (n = 1, 2.22%) (Sanchez et al., 2024).

### 3.6. The Levels of Prevention

The application of behavioral change theories or models across prevention levels, is presented as follows:

(a) Primary prevention (n = 27, 60%) (Barrett et al., 2018; Bestle et al., 2020; C. Chen et al., 2020; Cleary et al., 2014; Conroy, 2022; Demetriou & Bachner, 2019; Ek et al., 2018; Freyer-Adam et al., 2022; Frost et al., 2022; Godoy-Izquierdo et al., 2023; Gul et al., 2019; Jolly et al., 2018; Lackinger et al., 2015; Larsen et al., 2020; Latomme et al., 2021; Lubans et al., 2016; Lyon et al., 2024; Nakamura et al., 2017; Patel et al., 2019; Perry et al., 2023; Pond et al., 2019; Rashid et al., 2022; Sanchez et al., 2024; Stea et al., 2016; Wallbank et al., 2019; K. Wang et al., 2022; Yoong et al., 2022);
(b) Secondary prevention (n = 9, 20%) (Abidi et al., 2016; Ara et al., 2019; Caudwell et al., 2016; W. Chen et al., 2016; Delea et al., 2019; Frost et al., 2022; Howlett et al., 2017; Mat Said et al., 2021; Murawski et al., 2018);
(c) Tertiary prevention (n = 9, 20%) (Dobson et al., 2016; Haug et al., 2014; Kamal et al., 2016; Krämer et al., 2022; Larkin et al., 2017; Mâsse et al., 2020; Pakpour et al., 2022; Stea et al., 2016; Ting et al., 2018).

## 4. Discussion

Health behavior change plays a critical role in promoting and enhancing the overall health and well-being of individuals and communities (Anderson et al., 2004; Glanz & Bishop, 2010; Lippke et al., 2012; Michie et al., 2018b; Noar et al., 2008; Ory, 2002; Rimer et al., 2001; Zhang et al., 2024). It is a key contributor to disease prevention, significantly reducing morbidity and mortality by targeting and modifying health-related behaviors (Caron et al., 2023; Keller et al., 2021; Lippke et al., 2012; Rimer et al., 2001; Rubinelli & Diviani, 2020; Zhang et al., 2024). Encouraging healthier behavior patterns lies at the core of many health promotion interventions, programs, and policies aimed at fostering a healthier lifestyle and improving individual well-being (Bestle et al., 2020; Caron et al., 2023; C. Chen et al., 2020; Ek et al., 2018; Gul et al., 2019; Latomme et al., 2021; Lippke et al., 2012; Michie et al., 2018b; Patel et al., 2019; Steinmetz et al., 2016; K. Wang et al., 2022; Zhang et al., 2024).Health promotion interventions often use and incorporate specific behavioral theories and models, since these provide essential theoretical frameworks to understand, explain, predict, and influence health-related behaviors (Glanz & Bishop, 2010; Hagger & Weed, 2019; Lippke et al., 2012; Paek et al., 2010; Prestwich et al., 2015; Rimer et al., 2001; Silva et al., 2024; Steinmetz et al., 2016; J. Wang et al., 2025). Over the past few decades, the scientific literature has advanced health promotion strategies and highlighted the importance of applying effective behavioral change theories and models to achieve positive health outcomes (Anderson et al., 2004; Caron et al., 2023, 2023; Fishbein & Yzer, 2003; Hagger & Weed, 2019; Michie et al., 2018b; Morabia & Costanza, 2010; Steinmetz et al., 2016; Zhang et al., 2024). Furthermore, recent research has increasingly focused on translating health promotion findings—particularly those targeting behavior change—into practice (Glanz et al., 2008a; Morabia & Costanza, 2010; Rimer et al., 2001; Steinmetz et al., 2016; Tate et al., 2016; Zhang et al., 2024). While health promotion interventions targeting behavior change have proven rather effective, there remains a need for more systematic, comprehensive, transparent, and sustainable research efforts (Glanz & Bishop, 2010; R. Glasgow et al., 2004; Hagger & Weed, 2019; Michie et al., 2018a; Morabia & Costanza, 2010).

Understanding why some health promotion interventions succeed while others fall short is essential for making better-informed intervention decisions targeting health-related behaviors (Anderson et al., 2004; R. E. Glasgow & Emmons, 2007; Michie et al., 2018a; Michie & Johnston, 2012). To deepen this understanding, the theoretical components that predict behavior change must be more clearly defined, particularly in relation to the motivations and intentions driving such change (Lippke & Ziegelmann, 2008). Moreover, theory-based interventions should systematically derive specific behavior change techniques from the underlying theory and apply them in practice to achieve the intended outcomes effectively (Lippke & Ziegelmann, 2008).

This rapid review identifies health promotion interventions grounded in behavioral change theories and models, targeting diverse populations and addressing a wide range of topics. It provides a comprehensive overview of the evolving field of health promotion interventions focused on behavior change research and practice. Specifically, it emphasizes the importance of theoretical foundations in behavior change, particularly regarding the implementation and sustainability of health promotion interventions (Michie et al., 2018b; Perry et al., 2023; Sanchez et al., 2024; Ting et al., 2018).

The study revealed that combining behavioral change theories or models was more common than applying them individually (Abidi et al., 2016; Abildsnes et al., 2017; Caudwell et al., 2016; C. Chen et al., 2020; W. Chen et al., 2016; Conroy, 2022; Delea et al., 2019; Demetriou & Bachner, 2019; Ek et al., 2018; Gardner et al., 2014; Howlett et al., 2017; Jolly et al., 2018; Kamal et al., 2016; Larsen et al., 2020; Latomme et al., 2021; Lubans et al., 2016; Lyon et al., 2024; Mâsse et al., 2020; Mat Said et al., 2021; Nakamura et al., 2017; Patel et al., 2019; Perry et al., 2023; Pond et al., 2019; Rashid et al., 2022; Sanchez et al., 2024; Stea et al., 2016; Wallbank et al., 2019; Yoong et al., 2022). Studies confirm that integrating multiple behavior change theories and models is commonly employed in health promotion interventions (Bohlen et al., 2020; Prestwich et al., 2015; Richards & Johnson, 2014). However, it remains to be seen how effective the application of multiple theories and models will be in the future (Diep et al., 2014; Gourlan et al., 2016; Hagger & Chatzisarantis, 2014; Pound & Campbell, 2015; Prestwich et al., 2015).

Regarding the individual application of behavioral change theories or models, the Theory of Planned Behavior (Cleary et al., 2014; Larkin et al., 2017; Ting et al., 2018), the Social Cognitive Theory (Barrett et al., 2018; Bestle et al., 2020; Murawski et al., 2018), and the Health Action Process Model (Godoy-Izquierdo et al., 2023; Haug et al., 2014; Krämer et al., 2022) were used most frequently. Less commonly applied were the Transtheoretical Model (Freyer-Adam et al., 2022; Pakpour et al., 2022), Emerging Models (Gul et al., 2019; Lackinger et al., 2015), the Health Belief Model (K. Wang et al., 2022), the COM-B Model, (Frost et al., 2022), the Behavior Change Communication (Ara et al., 2019), and Behavior Change Techniques (Dobson et al., 2016). A recent systematic review identified the Health Belief Model, Theory of Planned Behavior, and Protection Motivation Theory as the most commonly cited models (Weston et al., 2020).

Furthermore, this rapid review encompassed a wide range of health promotion topics to which these theories or models were applied, including key categories such as main risk factors (e.g., physical activity, nutrition, combined physical activity and nutrition, alcohol use, and alcohol and tobacco use), prevention of non-communicable diseases, mental health prevention and promotion, and prevention of communicable diseases. Health promotion interventions often utilize a variety of theoretical frameworks to effectively address diverse health topics (Bully et al., 2015; Panagopoulou et al., 2011; Prochaska et al., 2008; J. Wang et al., 2025; Zhang et al., 2024).

Regarding the application of behavioral change theories or models in terms of levels of influence, based on McLeroy’s Socio-Ecological Model (McLeroy et al., 1988), it was found that most of the applied behavioral change theories or models targeted the intrapersonal level of influence. In terms of prevention levels, the majority of studies focused on primary prevention, while secondary and tertiary prevention were less frequently targeted (Fishbein & Yzer, 2003). Health promotion interventions using behavioral change theories or models, primarily targeting the individual level, aim to modify personal behaviors and perceptions to improve health outcomes (Becker et al., 2020; Davis et al., 2015, 2015; Rubinelli & Diviani, 2020; J. Wang et al., 2025). The literature suggests that exploring the boundaries of these theories, expanding research methods, and testing them with relevant outcomes could facilitate their translation to the interpersonal and community levels (Davis et al., 2015; Lippke & Ziegelmann, 2008; Mermelstein & Revenson, 2013; J. Wang et al., 2025).

In conclusion, the findings emphasize the importance of multi-level approaches to health promotion interventions, which could propel the field of behavior change in new directions and help bridge the gap between research and practice (R. Glasgow et al., 2004; Hagger & Weed, 2019; Michie et al., 2018b; J. Wang et al., 2025). This study provides valuable insights for researchers and practitioners in designing, implementing, and evaluating theory-driven interventions moving forward (R. Glasgow et al., 2004; Steinmetz et al., 2016; Willmott & Rundle-Thiele, 2021).

## 5. Strengths and Limitations of the Study

Although the rapid review methodology employed in this study offers several advantages, it also has certain limitations. One of the main advantages of this rapid review is its ability to gather significant information on health promotion interventions. Summarizing and synthesizing recent relevant literature is crucial for understanding the latest efforts and evidence surrounding these interventions. Additionally, this rapid review enabled a focused investigation into the field, highlighting both advancements and challenges in the existing literature. It also addressed the increasing methodological rigor in health promotion interventions and the use of theory-based approaches in these efforts.

Regarding the limitations of the present rapid review, it is important to note that, due to the nature of this methodology, a comprehensive analysis of all relevant aspects of the subject was not possible. Additionally, the rapid review methodology may have limitations in terms of the depth of analysis of health promotion interventions targeting behavior change. To strengthen the generalizability, robustness, and effectiveness of such interventions, further studies are needed. In light of these limitations, additional research in the field of health promotion targeting behavior change is essential. This will help bridge the gap between health promotion research and practice, offering valuable insights for public health policy decisions. Ultimately, this rapid review provides a focused and efficient investigation of the existing literature, highlighting key insights into the importance of applying behavioral change theories and models in health promotion interventions.

## 6. Conclusions

This study employed a rapid review methodology to inform future research efforts by exploring the application of behavioral change theories and models in health promotion interventions, which address a wide range of topics and target diverse populations. Overall, the findings highlight the urgent need for a more comprehensive and systematic investigation into the widespread application of health promotion interventions targeting behavior change in practice. Moving forward, future research should focus on the long-term potential of integrating these findings into national health policies for diverse populations. In summary, there is a critical need to bridge the gap between behavioral health promotion research and its practice, ensuring the translation of research into practice. To achieve this, comprehensive, theory-based, and evidence-driven efforts are essential to enhance the efficacy, effectiveness, and widespread dissemination of these studies.

## Figures and Tables

**Table 1:** Summary of inclusion and exclusion criteria based on the PICOS framework.

		Inclusion Criteria	Exclusion Criteria
P	Populations/participants	All ages	None
I	Interventions	Based on behavioral change theories/models	Not based on behavioral change theories/models
C	Comparators	With or without comparison group	None
O	Outcome	Main outcome: health behavior change	None
S	Study design	Studies with sufficient and efficient reporting on methodological issues related to the choice and application of behavioral theories/models	Studies with no sufficient and efficient reporting on methodological issues related to the choice and application of behavioral theories/models

**Table 2:** A summary of the included studies.

Study	Subject	Theory/Model	Level of Influence	Level of Prevention	Study Design	N	Target Group	Aim	Intervention Description
Cleary et al. (2014)	Sun protection	Theory of Planned Behavior	Intrapersonal	Primary	RCT	420	Adults aged 18+	Evaluates an intervention to improve sun protection	Intervention group: online session Control group I: only information Control group II: nothing at all
Gardner et al. (2014)	Physical activity	Habit Formation Model Behavior Change Techniques	Intrapersonal	Primary	RCT	120	Adults aged 60–74 inactive or moderately inactive	Evaluates an intervention to reduce prolonged sedentary behavior	Intervention group: booklet and motivational text about light-intensity physical activity Control group: fact sheet outlining physical activity and sedentary behavior recommendations
Haug et al. (2014)	Alcohol and tobacco use	Health Action Process Model	Intrapersonal	Tertiary	Cluster RCT	Classes 1.350	University students who smoke and consume alcohol	Evaluates an intervention for smoking cessation and reduced alcohol consumption	Intervention group I: online session; telephone messages; messages for smoking cessation; option to enroll in a more intensive smoking cessation program Intervention group II: messages for smoking cessation and option to enroll in a more intensive smoking cessation program
Lackinger et al. (2015)	Physical activity	Logic model	Intrapersonal	Primary	Cluster CT	8 communities; 194	Adults aged 30–65 in health resorts with insufficient levels of physical activity	Evaluates an intervention for increasing physical activity	Intervention group: sessions of physical exercise Control group: leaflet about physical activity
Abidi et al. (2016)	Alcohol use	Behavior Change Wheel COM-B model (Capability, Opportunity, and Motivation) Theoretical Domain Framework	Intrapersonal	Secondary	Cluster RCT	44 practices	Adults aged 18+ (General practitioners and patients)	Evaluates an intervention for reducing alcohol consumption	Intervention group: online sessions (ASBI) Control group: care as usual
Caudwell et al. (2016)	Alcohol use	Theory of Planned Behavior Self-determination Theory Behavior Change Techniques	Intrapersonal	Secondary	RCT	196	University students drinking before going out (pre-drinking)	Evaluates an intervention for reducing pre-drinking	Intervention group I: online sessions for autonomy support Intervention group II: online sessions for implementation intention Intervention group III: combined the autonomous motivation and the intervention intention Control group: nothing at all
W. Chen et al. (2016)	Work environment health and safety issues	Health Belief Model Social Cognitive Theory Theory of Planned Behavior Andersen’s behavioral model of health services use	Intrapersonal Interpersonal	Secondary	Cluster RCT	60 companies	Adults aged 18+ Migrant workers in small or medium-sized companies	Evaluates an intervention to promote the use of personal protective equipment (PPE)	Intervention group Ι: Occupational health education for managers and occupational health personnel, including a lecture on general health education and occupational health messages (mHealth) Intervention group ΙΙ: The same intervention, plus peer education Control group: nothing at all
Dobson et al. (2016)	Diabetes	Behavior Change Techniques	Intrapersonal	Tertiary	RCT	1000	Adolescents aged 16+ with poor diabetes management	Evaluates an intervention to improve glycemic control	Intervention group: automated text messages for self-management support Control group: usual care
Kamal et al. (2016)	CVD	Social Cognitive Theory Health Belief Model Behavior Change Communication	Intrapersonal Interpersonal	Tertiary	RCT	200	Adults aged 18+ with a history of vascular disease	Evaluates an intervention to improve medication adherence and health literacy	Intervention group: SMS reminders customized individual prescription Control group: usual care
Lubans et al. (2016)	Physical activity	Social Cognitive Theory Self-determination Theory RE-AIM Model	Intrapersonal Interpersonal Community	Primary	Cluster RCT	32 Classes-640	Adolescents Aged 9–16	Evaluates an intervention for increasing physical activity	Intervention group: interactive seminars, structured physical activity programs, lunch-time fitness sessions, and web-based smartphone apps Control group: usual practice
Stea et al. (2016)	Weight management/obesity	Socio-ecological Model PRECEDE-PROCEED Model Self-determination Theory	Intrapersonal Interpersonal Community	Tertiary	CT	80	Children aged 6–10	Evaluates a family-based intervention to improve lifestyle habits in overweight and obese children	Intervention group: Family counseling, workshops focusing on family life regulation, nutrition classes, exercise groups, and practical learning sessions Control group: will receive the intervention after 6 months
Abildsnes et al. (2017)	Physical activity & nutrition	Transtheoretical Model Motivational interviewing Self-Determination Theory Logic model	Intrapersonal	Primary	RCT	118	Adults aged 18+ in healthy life centers	Evaluates an intervention for diet, sedentary behavior and physical activity	Intervention group: counseling sessions, behavior change interventions in groups, and an individual counseling session Control group: will receive the intervention after 6 months
Howlett et al. (2017)	Physical activity	Behavior Change Techniques Motivational interviewing COM-B Model	Intrapersonal	Secondary	Cluster CT	4 regions-1.500	Young Adults aged 16+ with one or more risk factors for CVD, insufficient physical activity and/or a mild to moderate mental health condition	Evaluates an intervention for increasing physical activity	Intervention group I: physical activity promotion through a booklet, consultations, phone calls, motivational messages, free exercise classes (standard delivery) Intervention group II: The same intervention, plus the support of exercise “buddies” (enhanced delivery)
Larkin et al. (2017)	Physical activity	Theory of Planned Behavior	Intrapersonal	Tertiary	RCT	40	Adults aged 18+ patients with rheumatoid arthritis	Evaluates an intervention for increasing physical activity	Intervention group: physical activity sessions Control group: leaflet about physical activity
Nakamura et al. (2017)	Nutrition	Health Belief Model Transtheoretical Model Social Cognitive Theory	Intrapersonal Interpersonal	Primary	RCT	1.500	Adults aged 30–59	Evaluates an intervention to promote vegetable intake	Intervention group: nutrition education via emails Control group: an e-mail for participation in a survey after 5 weeks
Barrett et al. (2018)	Physical activity	Social Cognitive Theory	Interpersonal	Primary	RCT	40	Adults aged 65+ living in nursing homes	Evaluates an intervention to improve physical activity and quality of life	Intervention group: physical activity sessions Control group: usual care
Ek et al. (2018)	Physical activity	Social Cognitive Theory Socio-ecological Model Behavioral Change Techniques	Intrapersonal	Primary	RCT	200	Adults aged 20–65	Evaluates an mHealth intervention to promote active transportation	Intervention group: behavior change support program combined with monitoring of active travel via the TRavelVU Plus app Control group: monitoring of active transport via the TRavelVU app
Jolly et al. (2018)	Nutrition	Behavior Change Wheel Behavior Change Techniques COM-B Model Logic Model	Intrapersonal	Primary	RCT	100	Women aged 16+ expecting their 1st child	Evaluates an intervention for feeding initiation before and after birth (ABA)	Intervention group: feeding helper approach Control group: usual care
Murawski et al. (2018)	Physical activity	Social Cognitive Theory	Interpersonal	Secondary	RCT	160	Adults aged 18+ with insufficient physical activity and undiagnosed sleep issues	Evaluates an mHealth intervention to improve physical activity and sleep quality	Intervention group: use of an app with immediate feedback on their goals Control group: will receive the intervention after 6 months
Ting et al. (2018)	Diabetes	Theory of Planned Behavior	Intrapersonal	Tertiary	RCT	180	Adults aged 18+ with type 2 diabetes	Evaluates an intervention for improving medication adherence	Intervention group: sessions for medication adherence Control group: questionnaire with the assistance of facilitator
Ara et al. (2019)	Nutrition	Behavior Change Communication	Intrapersonal	Secondary	Cluster RCT	13 unions-368 (children)	Households with economic problems	Evaluates a nutritional intervention for young children	Intervention group: food vouchers and micronutrient powder, counseling on child feeding, and water sanitation and hygiene Control group: health messages
Delea et al. (2019)	Sanitation	Theory of Triadic Influence Social Cognitive Theory Socio-ecological Model COM-B Model	Intrapersonal Interpersonal Community	Secondary	Cluster RCT	Regions-1500 households	Households with child/children in rural areas with hygiene issues	Evaluates an intervention to improve sanitation, hygiene, and mental well-being	Intervention group: sanitation and hygiene intervention Control group: FMoH’s existing CLTSH programming
Demetriou and Bachner (2019)	Physical activity	Self-determination Theory Social Cognitive Theory Youth Physical Activity Promotion Model (YPAPM)	Intrapersonal Interpersonal	Primary	Cluster RCT	Classes-600 (girls)	Girls aged 11–13	Evaluates an intervention for increasing physical activity	Intervention group: physical activities during lessons and leisure time Control group: will receive the intervention after 3 months
Gul et al. (2019)	Postpartum contraception	Integrated Behavior Model	Intrapersonal	Primary	RCT	840	Pregnant women aged 15–44 who are in their 1st or 2nd trimester	Evaluates an mHealth intervention for postpartum contraception and maternal health promotion	Intervention group I: voice and text messages Intervention group ΙI: interactive telephone counseling Control group: no additional phone-based support
Patel et al. (2019)	Nutrition	Transtheoretical Model Behavioral Change Communication	Intrapersonal	Primary	Cluster RCT	244 villages-2.051	20-week pregnant women with infant (until 12-month) in primary healthcare centers	Evaluates an mHealth intervention to reduce stunting in young children in rural areas	Intervention group: mobile phone-based behavior change communication about maternal and child health Control group: usual care
Pond et al. (2019)	Nutrition	Behavior Change Wheel COM-B Model Behavioral Change Techniques	Intrapersonal	Primary	Cluster RCT	18 Childcare services-355 (children)	Young children and their parents	Evaluates an mHealth intervention promoting healthy eating habits	Intervention group: mobile phone health intervention, app for parents about healthy nutrition and lunchbox content Control group: usual care
Wallbank et al. (2019)	Physical activity	Behavior Change Wheel COM-B Model	Intrapersonal	Primary	RCT	100	Women 50+ inactive	Evaluates an intervention for increasing physical activity	Intervention group: information session with email feedback, the use of a physical activity tracker (Fitbit) and a free trial session at the university sports facility Control group: will receive the intervention after 3 months
Bestle et al. (2020)	Nutrition	Social Cognitive Theory	Interpersonal	Primary	Cluster RCT	6 schools-160 (children)	Young children and their parents’ schools	Evaluates a family-based intervention for reducing the intake of sugar-rich discretionary food and drinks	Intervention group: health consultation with increased focus on discretionary food and drinks, a box of home-use materials, and peer-to-peer communication. Control group: usual care
C. Chen et al. (2020)	Physical activity	Behavioral Change Techniques Socio-Ecological model	Intrapersonal Interpersonal Community	Primary	Cluster RCT	18 offices-360 (employees)	Adults aged 18+ office employees	Evaluates an intervention to reduce sedentary behavior and increase physical activity	Intervention group: booklet, fitbit device, lottery-based incentives and team-based incentives Control group: nothing at all
Larsen et al. (2020)	Physical activity	Social Cognitive Theory Transtheoretical Model Motivational interviewing	Intrapersonal Interpersonal	Primary	RCT	154	Adults aged 70+ in community-dwelling	Evaluates an intervention for increasing physical activity	Intervention group: physical activity monitor (PAM)-based intervention and motivational interviewing Control group: will receive only the PAM-based intervention
Mâsse et al. (2020)	Weigh management/obesity	Behavior Change Techniques Social Cognitive Theory Player experience and need satisfaction theory Self-determination Theory ACUDO framework to promote engagement and enjoyment	Intrapersonal Interpersonal	Tertiary	RCT	Pediatric weight management clinics-200 (parent–child)	Children and adolescents aged 10–17 from pediatric weight management clinics	Evaluates an mHealth intervention to improve weight management and lifestyle outcomes	Intervention group: gamified app with health coaching and motivational interviewing techniques Control group: will receive the intervention after 3 months without the health coach
Latomme et al. (2021)		Behavior Change Wheel Theoretical Domain Framework Behavior Change Techniques COM-B Model	Intrapersonal	Primary	CT	102 (parent -child)	Young children and their fathers	Evaluates a family-based intervention to increase physical activity	Intervention group: interactive sessions and e-health component Control group: access to online session materials
Mat Said et al. (2021)	CVD	Self-management Model Social Cognitive Theory	Intrapersonal Interpersonal	Secondary	Cluster RCT	20 Health clinics132 (participants)	Adults aged 45+	Evaluates an intervention for improvement of stroke awareness	Intervention group: the standard clinical follow-up, informational leaflets and the Stroke Riskometer app Control group: informational leaflets
Conroy (2022)	COVID-19	Health Belief Model Theory of Planned Behavior Protection Motivation Theory Social Cognitive Theory	Intrapersonal Interpersonal	Primary	RCT	260	University students and teaching staff	Evaluates an intervention to promote preventive behavior for COVID-19	Intervention group Ι: outcome imagery exercise Intervention group IΙ: process imagery Intervention group IIΙ: outcome and process imagery Control group: face covering warning
Freyer-Adam et al. (2022)	Health Behaviors (tobacco use, alcohol use, physical activity, nutrition)	Transtheoretical Model	Intrapersonal	Primary	Single group intervention study	175	Adults aged 18–64, in general, hospitals	Evaluates an intervention to promote proactive behavior change among general hospital patients	Intervention group: modules for the lifestyle profile, physical activity, diet, alcohol and tobacco smoking
Frost et al. (2022)	Frailty	COM-B Model	Intrapersonal	Secondary	RCT	308	Adults aged 65+ in community-dwelling	Evaluates an intervention for mild frailty	Intervention group: Home Health service to maintain independence in older people with mild frailty Control group: care as usual
Krämer et al. (2022)	Depression	Health Action Process Model	Intrapersonal	Tertiary	RCT	128	Adults aged 18+ who meet criteria for major depressive episode	Evaluates a web-based intervention for behavioral activation	Intervention group: self-help modules (InterAktiv) targeting motivational and volitional competencies Control group: access in the InterAktiv, after the follow-up assessment
Pakpour et al. (2022)	Internet gaming disorder	Transtheoretical Model	Intrapersonal	Tertiary	RCT	206	Adolescents aged 13–18	Evaluates a mobile app-based intervention for the treatment of Internet gaming disorder (IGD)	Intervention group I: “HAPPYTEEN” app and consecutive sessions Intervention group II: a sleep hygiene intervention via the HAPPYTEEN app
Rashid et al. (2022)	Weight management/ obesity	Social Cognitive Theory Health Belief Model Transtheoretical Model	Intrapersonal Interpersonal	Primary	Cluster RCT	12 Preschools-460 (parents; children)	Preschool children and parents	Evaluates an intervention for weight management	Intervention group: interactive activities in the “MaCHeLclassroom”, while parents will have access to an online educational program and online parent–child activities at home Control group: parents will receive the link to the general health newsletters
Yoong et al. (2022)	Physical activity	Behavior Change wheel Behavior Change Techniques Theoretical Domains Framework COM-B Model Logic Model	Intrapersonal	Primary	RCT	100 ECEC services	Young children in Early Childhood Education and Care (ECEC) services	Evaluates an intervention to promote outdoor play in early childhood education and care services	Intervention group: opportunities for outdoor free play in young children Control group: usual care
K. Wang et al. (2022)	Oral health	Health Belief Model	Intrapersonal	Primary	Cluster RCT	26–36 childcare centers or kindergartens-518–628 (parent—child)	Children aged 18–30 months	Evaluates an mHealth intervention for promoting oral health habits in children	Intervention group: text messages to parents to promote oral health and control sugar intake in children Control group: a text message to parents
Godoy-Izquierdo et al. (2023)	Physical activity	Health Action Process Model	Intrapersonal	Primary	RCT	300	Women aged 45–65	Evaluates an intervention for increasing physical activity	Intervention group: supervised exercise program Control group I: active lifestyle Control group II: sedentary lifestyle
Perry et al. (2023)	Physical activity	Social Cognitive Theory Socio-ecological Model	Intrapersonal Interpersonal Community	Primary	Cluster RCT	20 towns -350–400	Adults aged 18+ living in rural areas	Evaluates an intervention for increasing physical activity	Intervention group I: “Step it up”: group-based walking program (standard approach) Intervention group II: a combined group-based walking plus civic engagement program (combined approach)
Lyon et al. (2024)	Social, emotional & behavioral development	Theory of Planned Behavior Health Action Process Approach Motivational interviewing	Intrapersonal	Primary	RCT	46 schools-276 (teachers)	Adults aged 18+	Evaluates an intervention for children’s social, emotional, and behavioral/mental health.	Intervention group: training includes volitional planning, attitudes, social norms, perceived behavioral control, action plans, and problem-solving Control group: training includes attitudes, social norms, perceived behavioral control, action plans, and problem-solving
Sanchez et al. (2024)	CVD	Behavior change wheel Theoretical domain framework RE-AIM Model	Intrapersonal Community	Primary	Cluster RCT	58 FPs	Family practitioners, Women aged 45+ and men aged 40+ with moderately elevated cholesterol levels	Evaluates an intervention for the primary prevention of cardiovascular disease	Intervention group: decision information strategy added to the non-reflective decision assistance Intervention group: reflective decision structure strategy added to the decision information and the non-reflective decision assistance strategies

Note: RCT = Randomized Controlled Trial; CT = Controlled Trial; N = number of participants; CVD: cardiovascular disease.

## Data Availability

No new data were created in this study.
